# Editorial

**DOI:** 10.1007/s12592-021-00384-3

**Published:** 2021-10-08

**Authors:** Werner Thole, Karin Böllert, Karin Bock, Rita Braches-Chyrek, Bernd Dollinger, Catrin Heite, Fabian Kessl, Holger Ziegler

**Affiliations:** 1 Technische Universität Dortmund, Dortmund, Deutschland; 2 Münster, Deutschland; 3 Dresden, Deutschland; 4 Bamberg, Deutschland; 5 Siegen, Deutschland; 6 Zürich, Schweiz; 7 Wuppertal, Deutschland; 8 Bielefeld, Deutschland

Schwierige Zeiten, die gegenwärtig zu gestalten sind. Nicht nur das gesellschaftliche, öffentliche und soziale Leben kann weiterhin aufgrund der pandemischen Situation nur eingeschränkt respektive unter spezifischen Bedingungen stattfinden, auch wissenschaftliche Aktivitäten suchten – und fanden – neue Formate. Das Publizieren von wissenschaftlichen Texten, so könnte angenommen werden, ist noch am wenigsten von den Einschränkungen betroffen, realisiert sich doch die Herstellung von Texten nicht – zumindest nicht hauptsächlich – in öffentlichen, sondern in privaten Räumen. Sicherlich sprechen gute Argumente für diese Sichtweise. Doch zugleich ist auch zu beobachten, dass Bücher, Zeitschriften, Herausgeber*innenbände und Sondereditionen verspätet oder gar nicht erscheinen. Auch diese Ausgabe der jetzt vorliegenden Zeitschrift *Soziale Passagen*, Heft 1 des Jahres 2021, erscheint mit einiger Verzögerung. Angefragte Beiträge erreichten die Redaktion verspätet, andere, zugesagte Beiträge wurden kurzfristig abgesagt. Dass das jetzt vorliegende Heft, erstmals in der über zehnjährigen Geschichte der Zeitschrift, ohne Beiträge zu einem Schwerpunktthema erscheint, ist eine Folgen der gegenwärtigen Situation. In der ersten Ausgabe der *Sozialen Passagen* werden Beiträge daher ohne Zuordnung zu einem Schwerpunktthema präsentiert. Nichtsdestotrotz freuen wir uns, dass wir freie Beiträge im Forum wie auch in den Forschungsnotizen zu ganz unterschiedlichen aktuellen Themen publizieren können.

Seit dem im Jahr 2019 beschlossenen Kohleausstieg ist die Lausitz als eine der drei bundesrepublikanischen Kohlereviere ins Bewusstsein einer breiteren Öffentlichkeit gerückt. *Alexandra Retkowski* geht auf Basis einer qualitativen Inhaltsanalyse von politischen, wirtschaftlichen und zivilgesellschaftlichen Positionspapieren Lausitzer Akteur*innengruppen in ihrem Beitrag der Frage nach, wie sich in diesen Stellungnahmen Forderungen und Zielvorstellungen eines sozialökologischen Wandlungsprozesses abbilden. Herausgestellt werden Gemeinsamkeiten zwischen den Perspektiven der Lausitzer Positionspapiere und den 17 Nachhaltigkeitszielen („sustainable development goals“, SDGs), und so wird das Profilbild eines möglichen Lausitzer Transformationsprozesses entlang der AGENDA 2030 entworfen.

*Kerstin Oldemeier* und *Alis Wagner* geben in ihrem Beitrag einen Einblick in die Online-Studie „Queeres Leben in Bayern“. Bei der Betrachtung der Diskriminierungserfahrungen wird die Existenz besonders vulnerabler Teilgruppen deutlich – sowohl innerhalb der Gruppe queerer Teilnehmer*innen als auch aus einem intersektionalen Blickwinkel. Die referierten Ergebnisse weisen auf eine geringe Inanspruchnahme von Beratungsangeboten und Anzeigenerstattung hin.

Ohne Adressat*innen respektive Klient*innen gäbe es keine Soziale Arbeit. Die Bezeichnung „Klient*in“ wirft jedoch die Frage auf, wer oder was genau damit gemeint ist und in welchem Verhältnis Soziale Arbeit zu Klient*innen steht. Dies scheint im Handlungsfeld der stationären Sozialen Altenarbeit besonders relevant. Dem Beitrag von *Grit Höppner *folgend, hat die fehlende Eindeutigkeit in der Zuschreibung des Klient*innenstatus zur Folge, dass der Auftrag der Sozialen Arbeit in diesem Handlungsfeld bisher nicht eindeutig definiert ist und die Einrichtung eines Sozialen Dienstes in Seniorenheimen keine Selbstverständlichkeit darstellt.

Die Forderung nach mehr Männern in sozialen Berufen wird seit einigen Jahren wiederkehrend in Politik, Medien und im Fachdiskurs aufgegriffen. Sie richtet sich dabei auf Tätigkeitsfelder, die alltagssprachlich als „Frauenberufe“ bezeichnet werden, da die sozialen Berufe sowohl historisch als auch aktuell einen hohen Frauenanteil aufweisen. *Susanna Booth *zeigt in ihrem Beitrag auf Basis einer wissenssoziologischen Diskursanalyse am Beispiel der kirchlichen Wohlfahrtsverbände, wie Gender im Diskurs um „mehr Männer in soziale Berufe“ auf der Ebene der Träger hervorgebracht wird.

Im öffentlichen Diskurs, in der sozialpädagogischen Praxis und Gewaltforschung lässt sich feststellen, dass häufig von einer Essenz des Phänomens „Gewalt“ ausgegangen wird. Auch in der empirischen Forschung zu Gewalt in Paarbeziehungen wird zumeist a priori gesetzt, was darunter gefasst wird. Wie Betroffene das Erlebte selbst deuten, steht hingegen nur sehr selten im Fokus. Basierend auf einer qualitativen Studie geht *Susanne Nef* daher der Frage nach, wie Personen, die häusliche Gewalt in Paarbeziehungen erfahren haben, die Gewalt und ihre Gewalterfahrungen deuten und wie diese Deutungen sozial hergestellt werden. Anhand einer theoretischen Modellierung wird aufgezeigt, dass Gewalt in Beziehungen nicht auf situative Dynamiken reduziert werden kann und nicht nur Gegenstand körperlicher Erfahrungen ist.

Beratung im Jobcenter unterscheidet sich von anderen Beratungssituationen unter anderem, so wird in einem weiteren Beitrag vorgetragen, durch den Abschluss einer Eingliederungsvereinbarung. Dies sind rechtsverbindliche Verträge zwischen Jobcenter und Arbeitsuchenden, die das Prinzip „Fördern und Fordern“ in der aktivierenden Sozialpolitik umsetzen sollen. Ob Eingliederungsvereinbarungen eine zentrale Rolle einnehmen, untersucht *Carolin Freier* unter den Bedingungen eines Feldexperimentes. Dabei zeigt sie, dass die Anlage des Beratungsgespräches als eher fordernd oder unterstützend und weniger durch den Abschluss oder Nichtabschluss der Eingliederungsvereinbarung beeinflusst wird.

Politische und professionelle Strategien kommunaler Sozialarbeitspolitik waren über Jahrzehnte eine disziplinäre und praktische Frage- und Aufgabenstellung für Thomas Olk und Hans-Uwe Otto. Mit dem in der Rubrik NACHGEFRAGT | WIEDERENTDECKT von *Peter Marquard* vorgestellten und reflektierten Aufsatz haben die beiden unter der Überschrift „Wertewandel und Sozialarbeit“ nach einem „Perspektivenwechsel in der Sozialarbeit“ gefragt und diesbezüglich in den 1980er Jahren aktuelle Trends der Entstaatlichung, der Professionalisierung und Selbsthilfe diskutiert. T. Olk und H.-U. Otto (1981, S. 100) registrieren eine Forderung zur „Rückkehr zu kleinen, überschaubaren Einheiten, nach dezentralen, unentgeltlichen und selbstbestimmten Formen der Dienstleistungsproduktion.“ Aktuell bleibt die Analyse zu den Chancen und Gefahren einer Kommunalisierung von Sozialpolitik, den Aufgaben einer Sozialen Arbeit vor Ort und den fachlichen wie politischen Herausforderungen für die Entwicklung der Profession.

Über die Auswirkungen der COVID-19-Pandemie auf die Fallarbeit der Jugendämter liegt bislang noch wenig empirisches Wissen vor. Gleichzeitig sind die konkreten Tätigkeiten der Fachkräfte mit Kindern, Jugendlichen und Familien sowie die Entscheidungspraktiken, etwa bezogen auf den Kinderschutz, bislang wenig erforscht. Die in der FORSCHUNGSNOTIZ von *Katharina Freres, Megan Benoit, Jana Posmek, Christopher Benkel, Nina Grüßert* und *Pascal Bastian* vorgestellte Studie basiert auf einem ethnografischen Forschungsprogramm. Aus einer relationalen Perspektive, die den Blick nicht alleine auf die jeweiligen Akteur*innen, sondern vielmehr auf deren Verbindungen und Vernetzungen untereinander richtet, sollen Verschiebungen des Netzwerks, in dem die Fälle üblicherweise bearbeitet werden, sichtbar gemacht und Praktiken, die sich als Bewältigungsstrategien auf die der veränderten Praxis fassen lassen, offengelegt werden. Im Fokus einer weiteren Forschungsnotiz von *Konstantin Rink, Tristan Gruschka, Udo Seelmeyer, Gudrun Dobslaw, Dominic Becking* und *Patrick Palsbröker *steht das Projekt Personalized Augmented Guidance for the Autonomy of People with Intellectual Impairments (PAGAnInI), das sich zum einen der technischen Entwicklung eines Smartphone-gestützten Lernsystems widmet, welches es Menschen mit kognitiven Beeinträchtigungen ermöglichen soll, sich weitgehend autonom im öffentlichen Raum zu bewegen. Zum anderen untersucht das Projekt den Implementierungsprozess dieser neuen digitalen Technologie in die Praxis der Behindertenhilfe. Das Projekt analysiert das Verhältnis von Technik, Akteur*innen und Adressat*innen in Bezug auf die Frage des Sozialpädagogischen und des professionellen Handelns.

Das ethnographische Forschungsprojekt von *Stephanie Pigorsch* bewegt sich in Settings veranstalteter Partizipationspraxis im Gemeinwesen. Es untersucht aus praxistheoretischer Perspektive, wie Artikulationen und Repräsentationen der Alltagsakteur*innen hervorgebracht und verhandelt werden. Reflektiert wird die Eingebundenheit der Sozialen Arbeit in eine von Widersprüchen gekennzeichnete Praxis. Hingewiesen wird so auf die Spannungsverhältnisse der professionellen Akteur*innen zwischen alltagsorientierter und institutionell geprägter Perspektive. Das Verbundvorhaben von *Werner Thole, Alexandra Engel, Alexandra Retkowski, Katja Drews, Jan Schametat, Heike Gumz* und *Julian Trostmann *zielt auf eine empirisch fundierte Darstellung der kulturellen Landschaften und insbesondere der Akteur*innenkonstellationen der kulturellen Bildung in drei als peripher bis sehr peripher gekennzeichneten Regionen. Konkret wird den Fragestellungen nachgegangen, welche Felder und Akteur*innen in welcher Form und mit welchen Angeboten den Kultursektor bespielen, welche Vernetzungsformen und Kooperationen bestehen, wie diese und wie auch die Praxis und die Praktiken hergestellt und mit welchen Sinnkonstruktionen von den engagierten Personen versehen werden. Eine weitere Forschungsnotiz von *Marius Metzger, Karin Stadelmann, Nicolette Seiterle* und *Annina Kienholz *setzt daran an, dass junge Mütter und deren Kinder im Entwicklungsverlauf besonders hohen Risiken, insbesondere einem Armutsrisiko ausgesetzt sind. Aus diesem Grund hat die Schweizer Großstadt Bern im Rahmen ihrer Armutsbekämpfungsstrategie ein Pilotangebot für junge Mütter konzipiert, welches deren Lebenssituation durch verschiedene Bildungs- und Begleitmaßnahmen langfristig verbessern soll. Mittels erstellter Prognosen zu zukünftig zu erwartenden Sozialhilfebezügen als zentralem Indikator für eine gelingende Armutsbekämpfung wird versucht, den Erfolg des Pilotangebotes vorauszusagen. Eine erste Erhebung bei der ersten Kohorte der Teilnehmerinnen des Angebotes zeigt nun, dass sich die Prognosen als stabil erweisen und die jungen Mütter tatsächlich finanzielle Eigenständigkeit erreichen können.

Dies ist das letzte Heft der Zeitschrift *Soziale Passagen*, das von der „Kasseler Redaktion“, Stephanie Simon und Werner Thole, verantwortet wird. Zukünftig wird die Zeitschrift von der „Wuppertaler Redaktion“, Sarah Henn und Fabian Kessl, koordiniert und verantwortet.

Wir wünschen eine anregungsreiche Lektüre und allen Leser*innen weiterhin gute Gesundheit in pandemischen Zeiten.

